# Joint and soft tissue injections in Irish primary care: a survey of GPs’ attitudes and practices

**DOI:** 10.3399/BJGPO.2022.0093

**Published:** 2023-03-08

**Authors:** James Farrell, Walter Cullen, John Broughan

**Affiliations:** 1 School of Medicine, University College Dublin, Dublin, Ireland; 2 Clinical Research Centre, School of Medicine, University College Dublin, Dublin, Ireland

**Keywords:** injections, intra-articular, physicians, general practice, primary health care

## Abstract

**Background:**

Musculoskeletal conditions are common in primary care, causing significant morbidity. Intra-articular and soft tissue corticosteroid injections are commonly performed by GPs internationally. It is unknown how commonly they are performed by GPs in the Republic of Ireland.

**Aim:**

To determine the frequency and type of joint and soft tissue injections performed by GPs in the Republic of Ireland and investigate factors affecting their use.

**Design & setting:**

A cross-sectional online questionnaire, which was based on previous international research, was devised for completion by GPs practising in the Republic of Ireland.

**Method:**

GPs were invited to electronically complete a questionnaire on their practices and attitudes regarding joint and soft tissue injections.

**Results:**

A total of 147 of 204 GPs (72.1%) had performed an intra-articular or soft tissue injection in the preceding year. GPs who were principals or partners, male, or worked in a rural or mixed urban and rural practice setting were more likely to perform these procedures. The most common injection sites were the shoulder and knee. Participants were confident about performing joint and soft tissue injections. It was found that 80.4% had received prior training in this treatment modality, most commonly during their GP training programme. A prolonged wait for secondary care intervention, symptom duration, and symptom severity were factors that increased the likelihood of performing injections. Difficulty maintaining skills and medicolegal concerns were common barriers to performing joint and soft tissue injections.

**Conclusion:**

Most GPs surveyed carried out joint and soft tissue injections, most commonly injecting the shoulder and knee. Irish GPs experience many of the same barriers to performing intra-articular injection as experienced internationally.

## How this fits in

This study has found that joint and soft tissue injections are commonly performed in general practice in the Republic of Ireland, highlighting patterns of practice and attitudes to the use of this intervention. Most GPs have received training in these procedures through a variety of means. Factors influencing the use of joint and soft tissue injections are identified, which may inform future training or service provision initiatives.

## Introduction

Musculoskeletal conditions are a common cause for presentation in the general practice setting. They account for 11%–21%^1,2^ of presentations in primary care globally, resulting in significant demands on healthcare resources.^3–5^ In the US alone, musculoskeletal conditions (excluding low back and neck pain) represent the second most expensive group of conditions in terms of healthcare spending.^4^ More than 9.6% of men and 18.0% of women aged >60 years worldwide have symptomatic osteoarthritis.^3^ Musculoskeletal conditions are associated with significant morbidity, decreasing quality of life, and increasing treatment burden.^5,6^ Long-term oral non-steroidal anti-inflammatory drugs (NSAIDs) and codeine-containing analgesics, often used in such conditions, are associated with significant potential adverse effects.^7,8^ This patient cohort often present in later life with significant comorbidities,^9^ which have logistical and cost implications for possible surgical treatment.

Many common musculoskeletal presentations, including knee and glenohumeral osteoarthritis, adhesive capsulitis, and subacromial impingement, are amenable to intra-articular or soft tissue corticosteroid injection.^10,11^ Treatment with corticosteroid injection can relieve symptoms rapidly^12^ and safely,^13^ allowing rehabilitation work and potentially delaying or eliminating the need for oral analgesia or surgery. Joint injections are not publicly funded in the GP setting in the Republic of Ireland, and wait times for public orthopaedic outpatient clinics are ≥12 months in 35% of cases.^14^

Previous published literature on the use of intra-articular injections in primary care is limited to a number of studies in the single digits. Several survey-based studies found that a majority of primary care doctors in a British setting^15,16^ performed intra-articular injections but that a large proportion of the injections performed were carried out by a small group of the practitioners who performed the procedures regularly. The applicability of these studies to current practice is limited, however, by the fact that they were carried out 15–17 years ago. In other regions such as the US and Saudi Arabia, these procedures were found to be carried out less commonly.^17,18^ It is not known if the use of joint and soft tissue injections in the Republic of Ireland mirrors practice internationally. The authors are aware of one study of minor surgery in Ireland that mentioned the number of joint injections performed by participants,^19^ but as this study involved a small subset of practitioners who carried out large volumes of minor surgery, it was not intended to be applicable to the broader general practice population. An examination of current practices of Irish GPs may identify any barriers to this intervention being used where appropriate and identify any training gap that may need to be addressed.

## Method

### Study design and participants

A self-administered questionnaire was designed with reference to a literature review on the topic of joint and soft tissue injections in general practice. The included questions examined practitioner demographics, current practice in the performance of joint injections, prior training in this area, and barriers to the use of this intervention — areas that have been studied internationally. As a knowledge gap was identified regarding individual patient factors, which would increase the likelihood of a GP performing a joint or soft tissue injection, new questions were formulated for this section. This was done with reference to American Academy of Orthopaedic Surgeons (AAOS) guidelines for management of the sites found to be most commonly injected in the literature review,^20^ and refined following discussion with a small group of practising GPs.

Participants were GPs practising in the Republic of Ireland recruited by the following two means: an email invitation sent to a group of GPs in the Ireland east region whose contact details were publicly available; and recruitment via GP-specific online forums (GP Buddy and GP Forum), where users are verified GPs. All participants were furnished with a participant information leaflet and contact details of the lead investigator. The invitation stated that participants did not need to perform joint injections to take part in an effort to limit a bias towards GPs with an interest in this area. Participants completed the questionnaire using an online survey platform, Google Forms. Responses were collected between 9 February 2022 and 27 March 2022.

### Inclusion and exclusion criteria

GPs working in the Republic of Ireland were eligible for inclusion. Doctors working in general practice in a training capacity, GP registrars, were excluded.

### Questionnaire structure

The questionnaire consisted of three sections. The first section recorded practitioner age, career length, practice role, and practice setting. The second queried the number of injections performed in the preceding year, confidence in performing injections, and prior training in this area. The third section examined attitudes to factors that were facilitators and barriers to the use of joint and soft tissue injections. Where possible, answers were collected using pre-determined answer selection options. These included binary yes or no choices, choices of anatomical site for injection, and five-point Likert scales to assess attitudes to various topics. All responses were anonymous.

### Statistical analysis

Statistical analysis was carried out using Microsoft Excel and IBM SPSS Statistics (version 27). The characteristics of the participants were described using medians and interquartile ranges (IQR) for continuous variables, and numbers and proportions for categorical variables. The χ^2^ test for independence was used to test for a relationship between categorical variables. The Kruskal–Wallis test was used to test for a significant difference in continuous variables between independent groups. A *P*-value of 0.05 was used to determine statistical significance.

## Results

### Participant sample and demographics

A total of 209 responses were received, of which 204 (97.6%) were included in the final data analysis. One duplicate response was reported and excluded, as were two responses from GP registrars and one from a retired former GP. One instance of extreme outlying data was excluded.

As participants were in part recruited from online GP forum sites (*n* = 111), it was not possible to calculate an overall response rate. However, when recruiting via email, 375 emails were delivered to valid addresses, and 93 responders completed the survey (response rate 24.8%). Sample demographics are demonstrated in Supplementary Table S1. The mean age of responders was 49.2 years, while the median age was 48 years (IQR 41–58). The age of participants ranged from 31–77 years. The majority of participants were males (63.2%), with the majority working as GP principals or partners (79.9%), most commonly in urban (47.6%) and mixed urban and rural practices (38.7%).

### Use of joint injections

It was reported that 72.1% of participants had performed a joint or soft tissue injection in the preceding 12-month period (Table 1). The median number of injections performed by these participants was 20 (IQR 10–50) and the mean number was 44.1 (standard deviation [SD] 75.71). Of the participants who had not performed an injection in the preceding 12 months, 61.4% of this group had previously performed at least one. Of the total study group, 89.2% had previously performed a joint or intra-articular injection at some point in their career (see Supplementary Table S2). The performance of joint and soft tissue injections by demographic subgroups is demonstrated in Table 1.

**Table 1.** Participants who have performed joint or soft tissue injection in the past year (*N* = 147)

Demographic group	GPs who performed injections in the past year, *n* (% of that subgroup^a^)	χ^2^ test, *P* value	Median injections performed by injection group, *n*	Interquartile range	Kruskal–Wallis test, *P* value
**Sex**					
Male	109 (84.5)	25.86, <0.001	30.0	12.0–50.0	32.51, <0.001
Female	38 (51.4)		10.0	5.0-15.7	
Prefer not to say	0 (0)		0.0	0.0	
**Age, years**					
<45	60 (73.2)	37.30, 0.59	24.5	8.7–40.0	1.89, 0.387
45–55	45 (70.3)		20.0	10.0–40.0	
>55	42 (72.4)		20.0	10.0–60.0	
**Career length, years**					
<10	39 (75.0)	57.58, 0.68	16.0	5.0–40.0	4.89, 0.087
10–20	46 (73.0)		30.0	12.0–50.0	
>20	62 (69.7)		20.0	10.0–50.0	
**Practice setting**					
Urban	61 (62.9)	7.78, 0.02	20.0	10.0–40.0	2.10, 0.350
Rural	23 (82.1)		30.0	10.0–50.0	
Mixed urban and rural	63 (79.8)		27.0	10.0–50.0	
**Job role**					
GP principal or partner	121 (74.2)	2.13, 0.54	27.0	10.0–50.0	11.38, 0.010
GP assistant	19 (63.3)		16.0	8.5–35.0	
Locum	6 (66.7)		4.5	2.5–5.7	
Other	1 (50.0)		12.0	6.5–7.5	

^a^See Supplementary Table S1 for subgroup *N*-values.

A higher proportion of males than females had performed an injection in the past 12-month period, and this group reported a higher median number of injections performed. A higher proportion of GPs in rural and mixed urban and rural areas performed injections than their urban counterparts, as did a higher proportion of GP principals or partners compared with GPs with other job roles. Each of these groups reported higher median numbers of injections performed than their counterpart demographic groups. Observed differences did not vary to the same degree when comparing stratified groupings of career length and age, as noted in Table 1.

The differences in proportions of GPs who had performed an injection in the preceding 12 months was statistically significant for the groupings of sex and practice setting but not for the variables of GP job role, age, and career length. The difference in number of injections performed across groups was statistically significant for the variables of sex and job role. It was not statistically significant for the variables of practice setting, career length groupings, and age groupings (Table 1).

A small subset of participants performed a disproportionately large number of the total reported injections. Of the estimated 6480 injections reported by the 147 participants who injected in the preceding 12 months, 18 participants reported performing ≥100 injections in the preceding year, meaning that 12.2% of the active injecting group accounted for 53.7% of all reported injections (see Supplementary Table S3).

### Sites of injection

The most commonly injected anatomical sites are demonstrated in Table 2.

**Table 2.** Anatomical injection sites

Anatomical site injected	*n* (% of all participants)
Shoulder	168 (82.4)
Knee	159 (77.9)
Subacromial space	114 (55.9)
Lateral epicondyle	94 (46.1)
Greater trochanteric bursa	87 (42.6)
Small joints of hands or feet	58 (28.4)
Carpal tunnel	16 (7.8)
Plantar fascia	9 (4.4)
Ankle	6 (2.9)
Hand tendons	5 (2.5)
Sacroiliac joint	5 (2.5)
Other	4 (2.0)
Wrist	3 (1.5)
Acromioclavicular joint	2 (1.0)
Facet joint of spine	2 (1.0)

The most commonly injected sites were the shoulder, knee, and subacromial space. More than half of the study participants reported having previously injected one of these sites. Less commonly injected sites are noted in Table 2.

### Confidence in performing joint and soft tissue injections

Participants who reported performing joint injections were asked to report their confidence in performing such procedures on a Likert scale of 1 to 5. A high level of confidence of 4 or 5 out of 5 was expressed by 74.2%, while 13.2% expressed a low level of confidence, which was indicated by a 1 or 2 out of 5 score (see Supplementary Table S4).

### Prior training in joint and soft tissue injections

A majority of participants (80.4%) had previously completed at least one of a number of specified training opportunities in intra-articular injection. The most common form of training was practical training during a GP training programme. Other means of training are demonstrated in Table 3.

**Table 3.** Prior training received in joint and soft tissue injections

Type of prior training received	*n* (% of all participants)
Practical training during a GP training programme	87 (42.6)
Musculoskeletal medicine course	62 (30.4)
Joint injection course	50 (24.5)
Hospital-based musculoskeletal medicine rotation	41 (20.1)
Community musculoskeletal medicine rotation	3 (1.5)
None of the above	40 (19.6)

GPs who had received practical training during their GP training programme were significantly more likely to have performed an injection in the past year (80.4%) than those who did not receive this training (65.8%, χ^2^ = 5.317, *P* = 0.021) (see Supplementary Table S5).

### Facilitators and barriers to the use of joint and soft tissue injections

**Figure 1.**
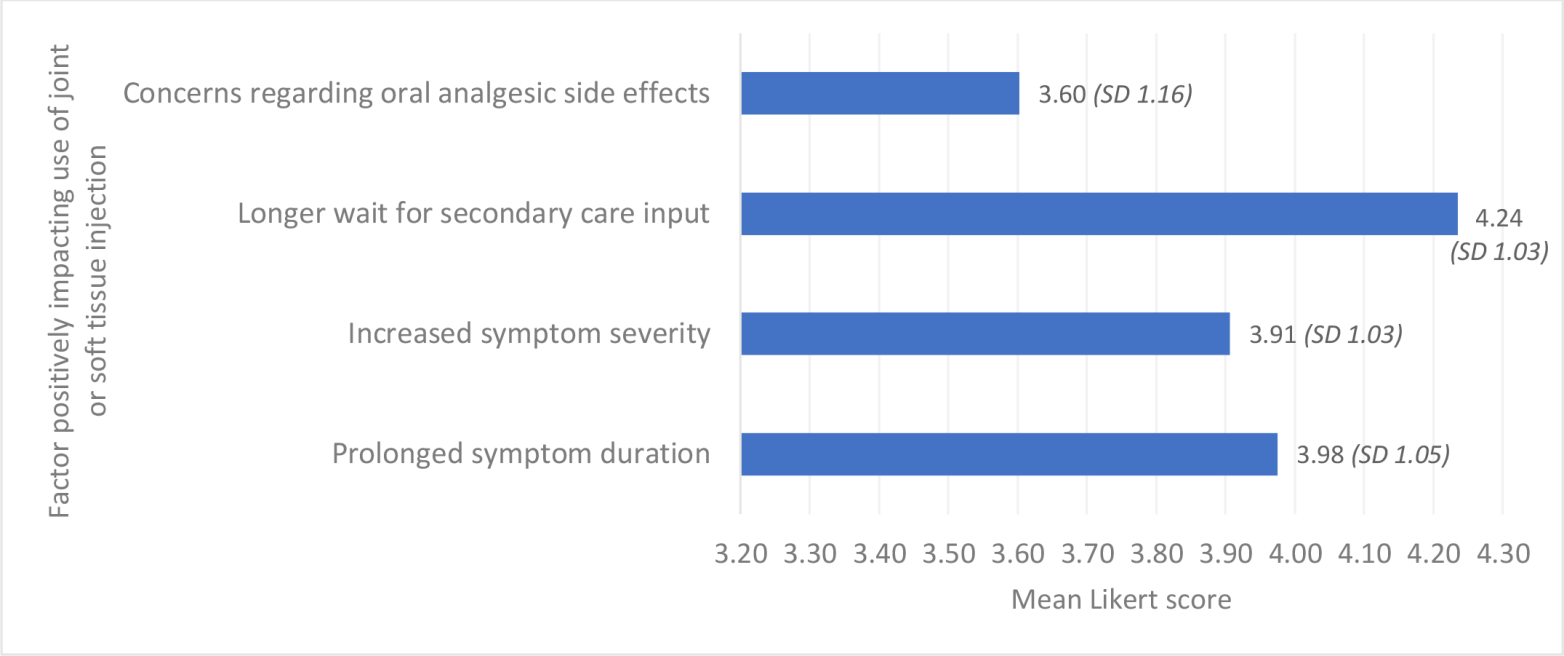
Facilitators of joint and soft tissue injection. SD = standard deviation.

The mean Likert scores for the factors examined are demonstrated in Figures 1 2. When asked about factors increasing the likelihood of performing injection in the primary care setting, a prolonged wait for secondary care input received the highest mean Likert score. Increased symptom severity and duration were also rated as being important (Figure 1).

**Figure 2.**
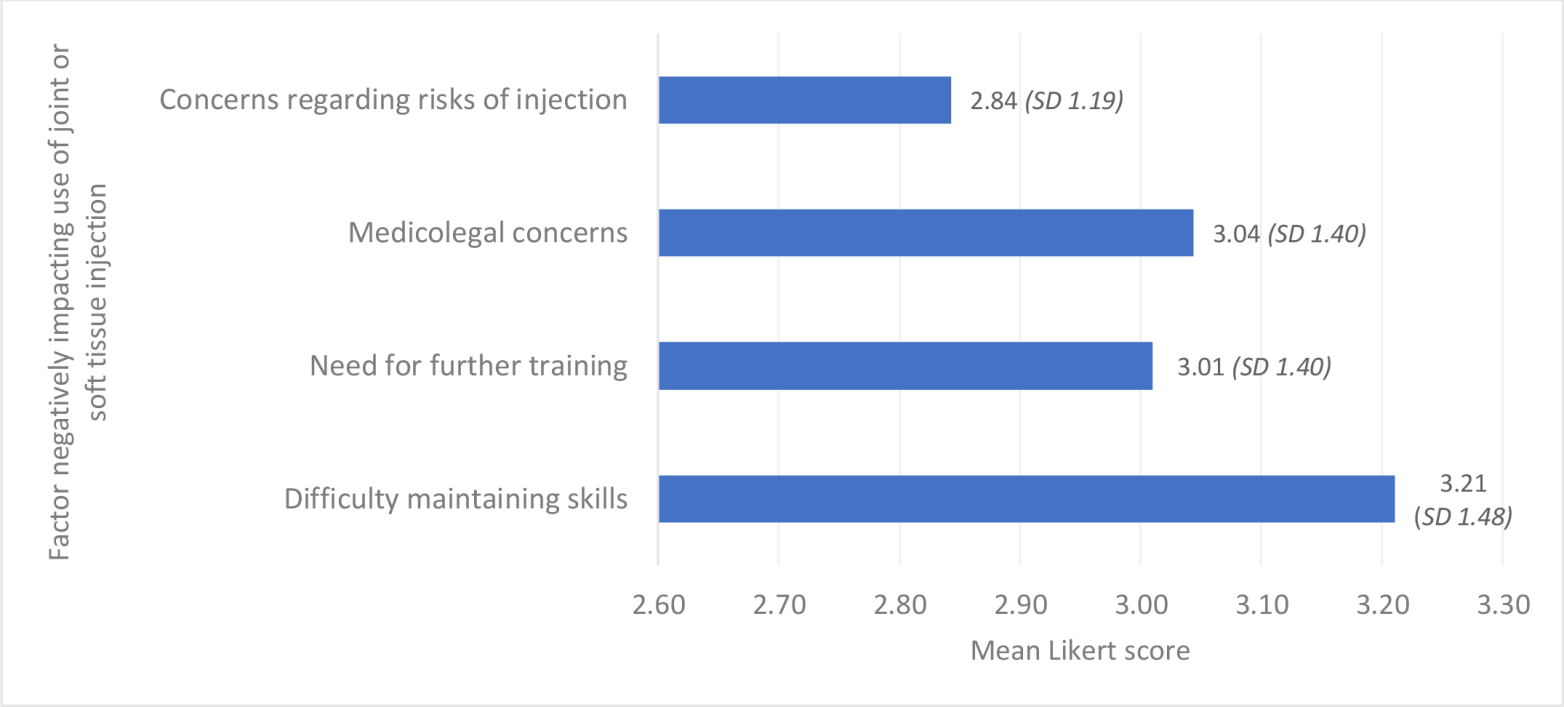
Barriers to joint and soft tissue injection. SD = standard deviation.

Difficulty maintaining injection skills was rated as the most important barrier to performing intra-articular and soft tissue injections. Medicolegal concerns were the second most important barrier cited (Figure 2).

## Discussion

### Summary

This study highlighted that joint and soft tissue injections were commonly performed by surveyed GPs in the Republic of Ireland. Male GPs, those in rural and mixed urban and rural practices, and GP principals or partners were more likely to perform these procedures. The most commonly injected anatomical sites were the shoulder, knee, subacromial space, and lateral epicondyle. GPs who performed joint injections were confident in doing so. Most GPs had received prior training in this treatment modality. A prolonged wait for secondary care intervention, symptom duration, and symptom severity were factors that increased the likelihood of performing injections. Difficulty maintaining skills, medicolegal concerns, and need for further training were common barriers to performing joint and soft tissue injections.

### Strengths and limitations

To the authors’ knowledge, this is the first study designed to investigate the use of joint and soft tissue injections by GPs in the Republic of Ireland. Based on the demographic characteristics observed, the survey sample broadly represented the makeup of the Irish GP population in terms of age and practice type.^21,22^ The number of participants (*N* = 204) was large enough to be meaningful. For context, there are approximately 3900 practising GPs in the Republic of Ireland,^21^ although this figure does not take into account differences in sessional working hours and roles in non-clinical or non-traditional settings.

The study design, drawing on existing literature in this area in the UK, allowed comparison to be made with previous research, while addressing the need for more up-to-date research in this area. In addition, the examination of participants' attitudes to patient factors in the use of joint and soft tissue injections shed light on an area that had not previously been formally studied.

This study had a number of potential limitations. As with opt-in survey-based studies more broadly, participants with an interest in this topic may have been more likely to complete the survey, reducing the broader applicability of the results. The study population was composed of a greater proportion of males (63.2%) compared with the national population of GPs (46%).^21^ Participants recruited by email invitation comprised of GPs based in the Ireland east region, and so this cohort may be over-represented.

### Comparison with existing literature

The findings of this study are consistent with similar studies in neighbouring countries. In studies carried out in the UK, a majority of GPs performed joint and soft tissue injections, with rates varying from 54%^15^ to 86%^16^ between studies. Internationally, while there was good recognition of the value of these procedures,^17^ the limited data would suggest that they are less commonly performed.^17,23^ In more than one study, being male^15,16^ or working in a rural or ‘mixed’ area were associated with performing soft tissue and joint injections.^15,23^ In the four studies that examined barriers to injection, a lack of training was consistently cited, as were an inability to maintain injection skills, diagnostic uncertainty, and medicolegal concerns.^15–18^ Each of these trends corresponded with the findings.

When examining self-reported number of injections performed, the findings of the present study were similar to those of Gormley *et al* in the UK^15^ in noting that a majority of injections were performed by a small subset of participants. The anatomical sites most commonly injected by Irish GPs were similar to that study and others.^15,16,18^ Fewer participants in the present study (24.5%) had attended a formal training course in joint injection, compared with a range of 50.6%^16^ to 73.0%^15^ in the UK-based studies. In keeping with these two UK studies, ‘difficulty maintaining skills’ ranked as the most common barrier to performing joint and soft tissue injections in the present study.

### Implications for research and practice

This study has highlighted questions for future research and areas for change in training and clinical practice. This study did not investigate non-clinical factors affecting use of injections, such as financial or logistical considerations, which could be explored in future research. Future research with broader recruitment may contextualise the applicability of the findings.

This study has highlighted that there is a potential need for greater training in this treatment modality. The most common form of training in joint and soft tissue injection was practical learning during a GP training programme, but a minority (42.4%) of responders had received this form of training, and 19.6% had not received any training at all in this intervention.

This study highlights a high level of provision of joint and soft tissue injections among surveyed GPs, albeit with the majority of injections being performed by a small subset of GPs. As an inability to maintain skills was the most commonly selected barrier to performing injections, increased availability of training programmes and systems of referral to GPs who perform large numbers of injections are two approaches that may improve availability of this treatment modality. As medicolegal concerns were also cited as a significant barrier, guidance from governing bodies in conjunction with greater training opportunities may help in this regard.

## Data

The dataset relied on in this article is available from the corresponding author on reasonable request.
